# Supplementary figures and images for: Cross-talk between microglia and neurons regulates HIV latency
Source: PLoS Pathog. 2019 Dec 30;15(12):e1008249. doi: 10.1371/journal.ppat.1008249 (PMC6953890; doi:10.1371/journal.ppat.1008249)

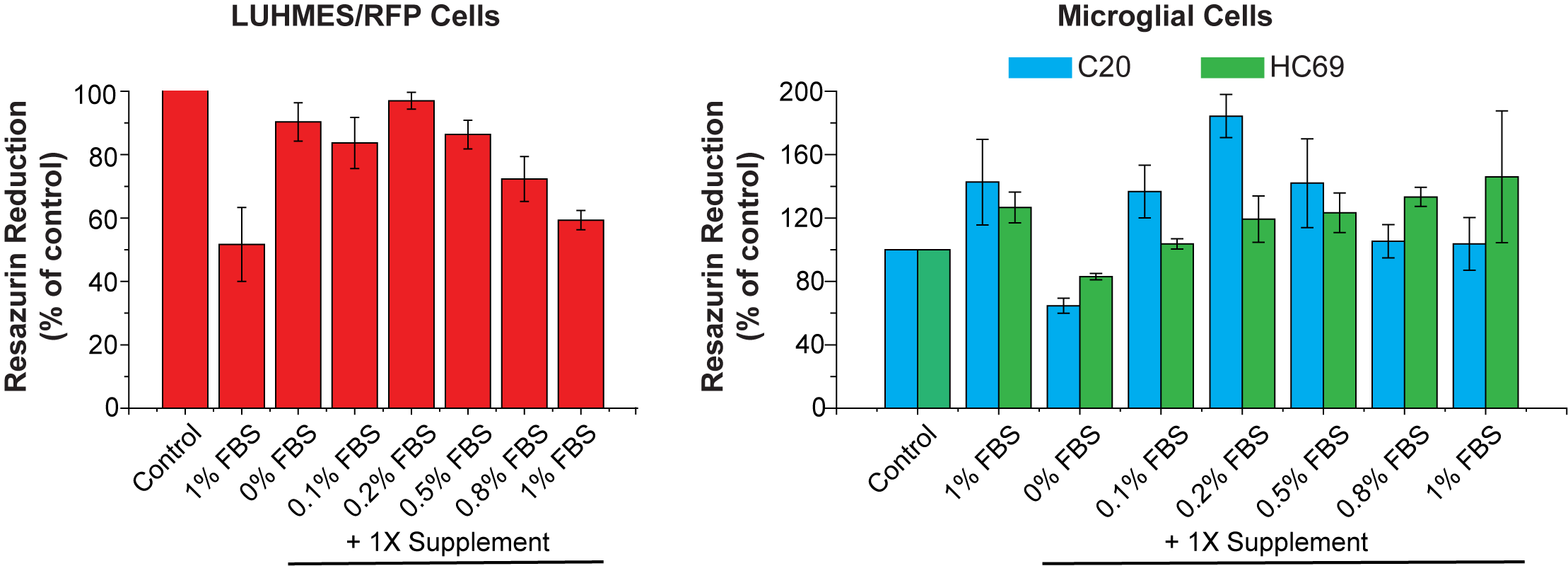

Supplement: S1 Fig — LUHMES-derived neurons (red) and microglial cells, C20 (blue) and HC69 (green), were independently cultured in the presence of the indicated medium formulations (X-axis) and cell viability (Y-axis) was measured by the resazurin method. (TIF) [file ppat.1008249.s001.tif]

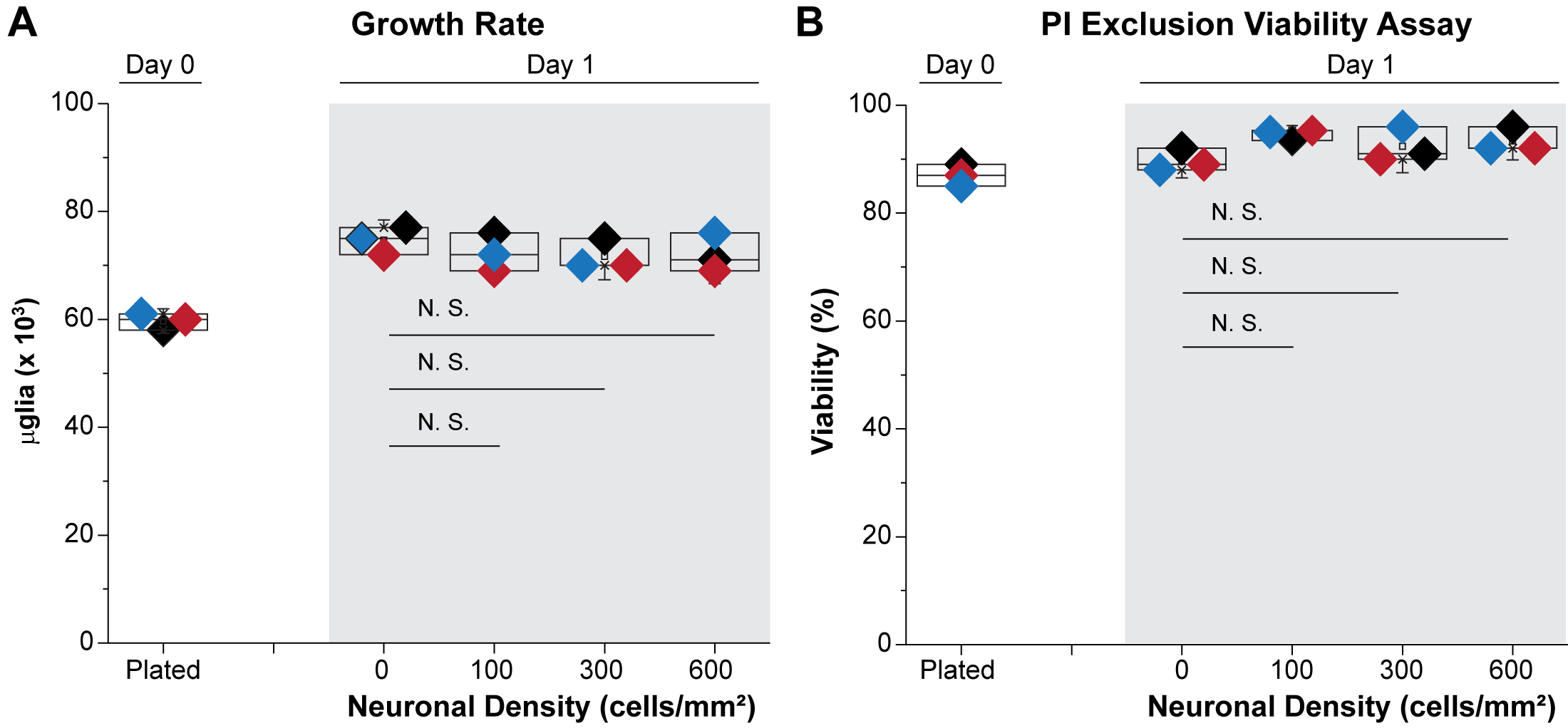

Supplement: S2 Fig — (A) Growth rate. 60,000 hμglia/HIV HC69 cells were plated in the presence of increasing density of LUMHES-derived neurons (X-axis). After 24 h (short-term), neurons were killed with 0.25% trypsin for 30 seconds, and washed away with PBS prior to further trypsinization for 5 minutes to recover microglial cells. Cells were counted (Y-axis). (B) PI exclusion assay for measuring viability (Y-axis; right panel). N.S.: not significant. (TIF) [file ppat.1008249.s002.tif]

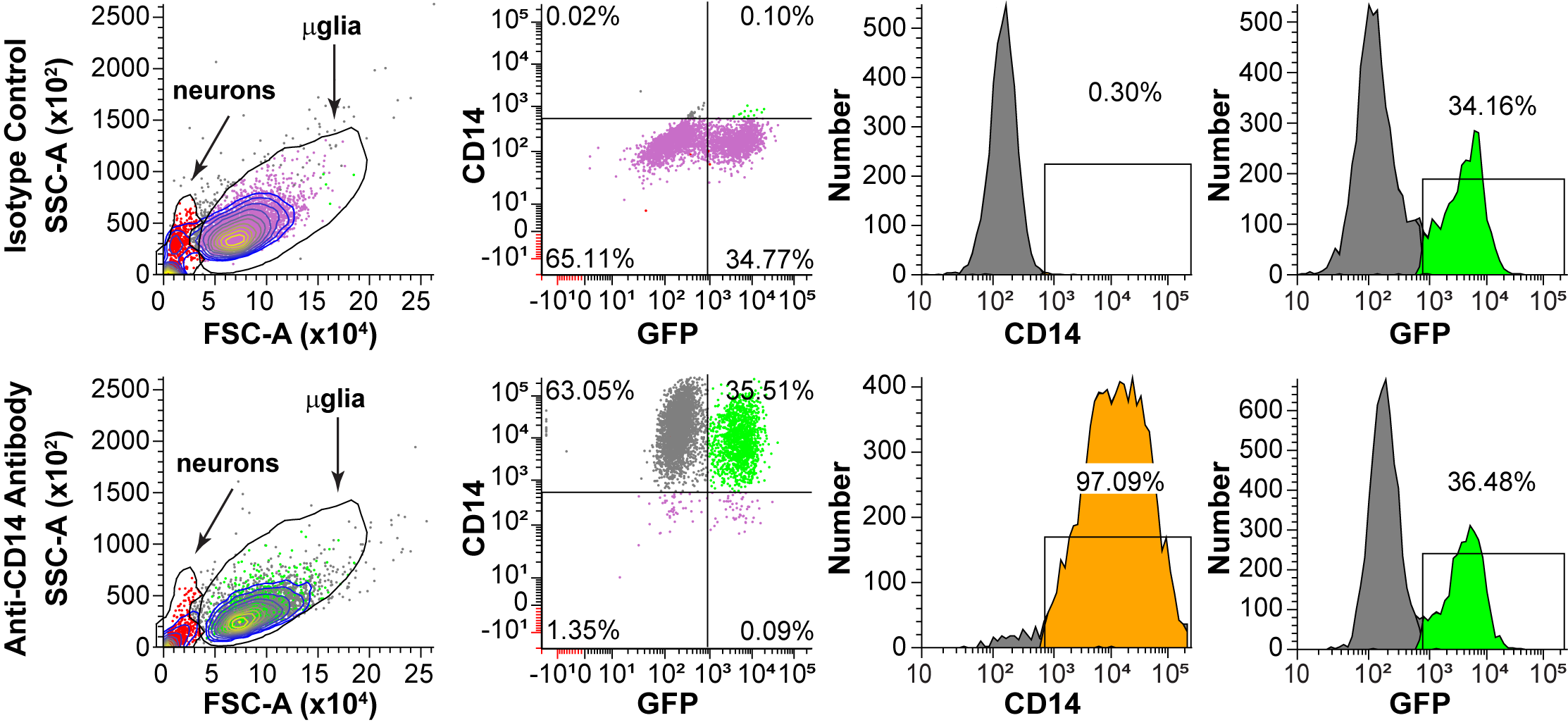

Supplement: S3 Fig — Flow cytometry profiles representing single cultures. The distinct populations of HC69 (μglia) and neuronal cells are indicated on the far-left flow cytometry profiles. Top flow cytometry profiles represent cells bound to isotype control; bottom profiles represent cells bound to anti-CD14 antibody. Anti-CD14 bound population is shown on the Y-axis, and GFP-expressing cells are shown on the X-axis in the CD14 vs. GFP graphs. The population of CD14-expressing cells is shown in orange and the populations of GFP-expressing cells are shown in green. (TIF) [file ppat.1008249.s003.tif]

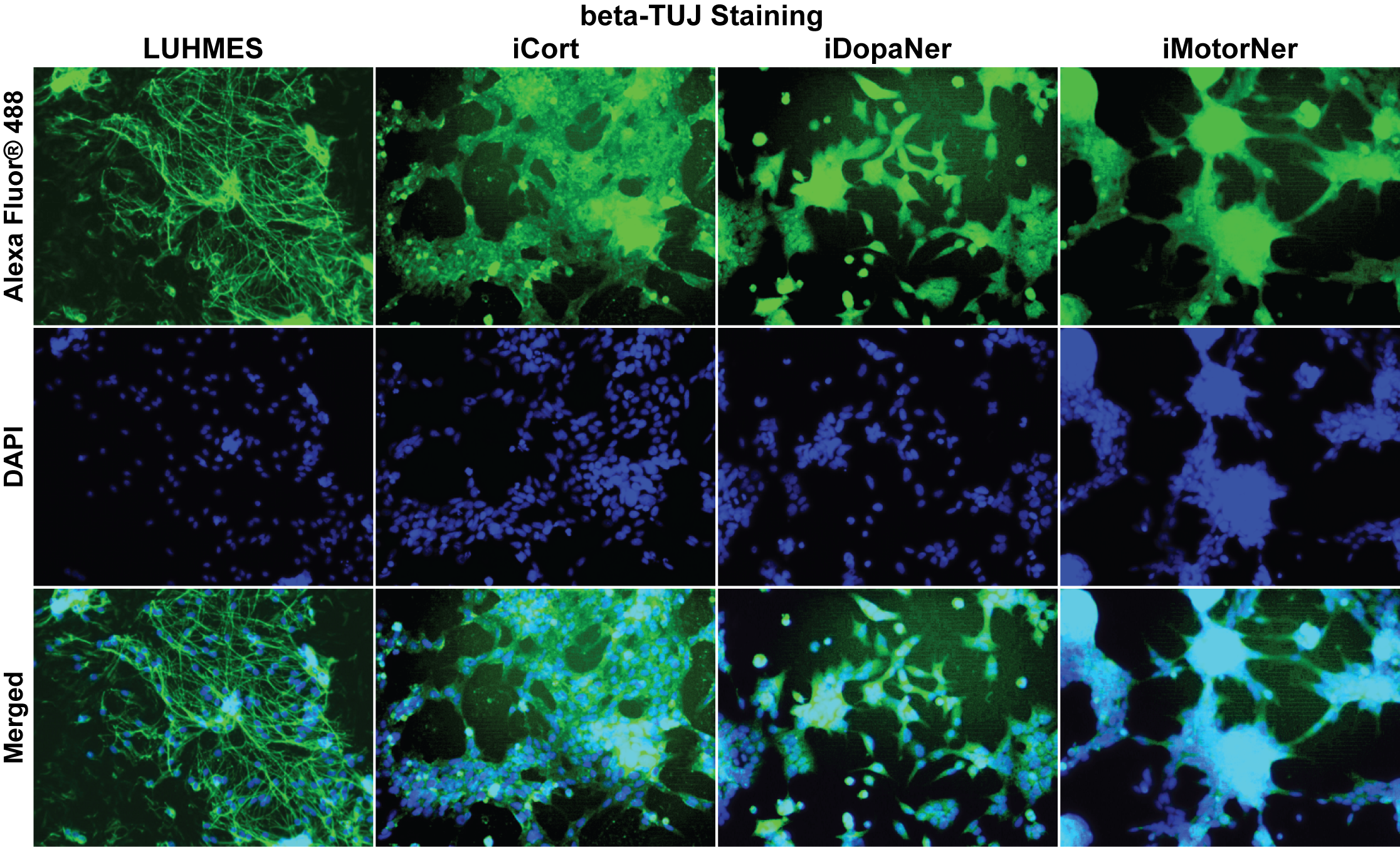

Supplement: S4 Fig — LUHMES- and iPSC-derived neurons were stained with antibody against beta-TUJ (green). Alexa Fluor 488 anti-rabbit was used as secondary antibody. DAPI (blue) indicates nuclear staining. (TIF) [file ppat.1008249.s004.tif]

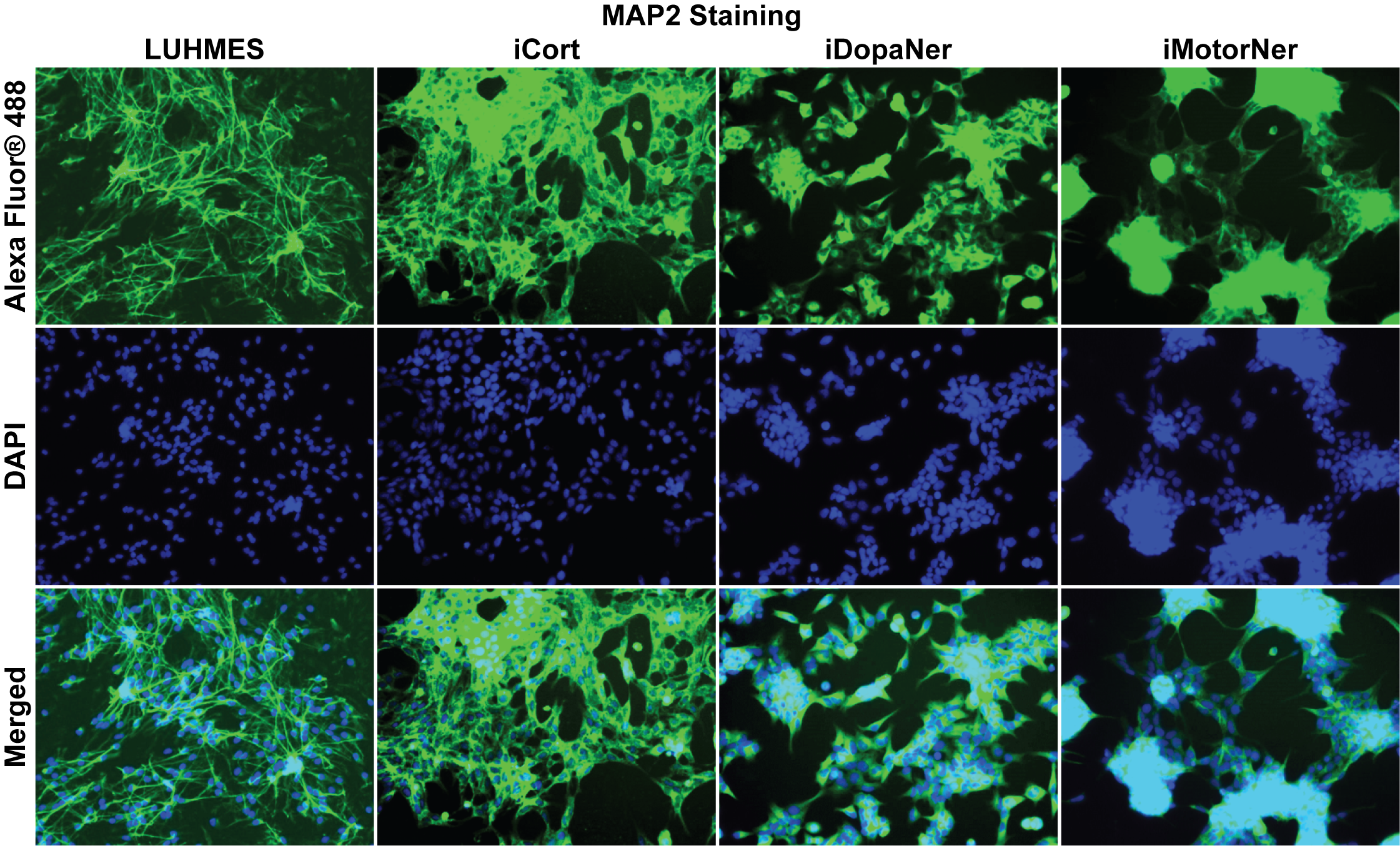

Supplement: S5 Fig — LUHMES- and iPSC-derived neurons were stained with antibody against MAP2 (green). Alexa Fluor 488 anti-rabbit was used as secondary antibody. DAPI (blue) indicates nuclear staining. (TIF) [file ppat.1008249.s005.tif]

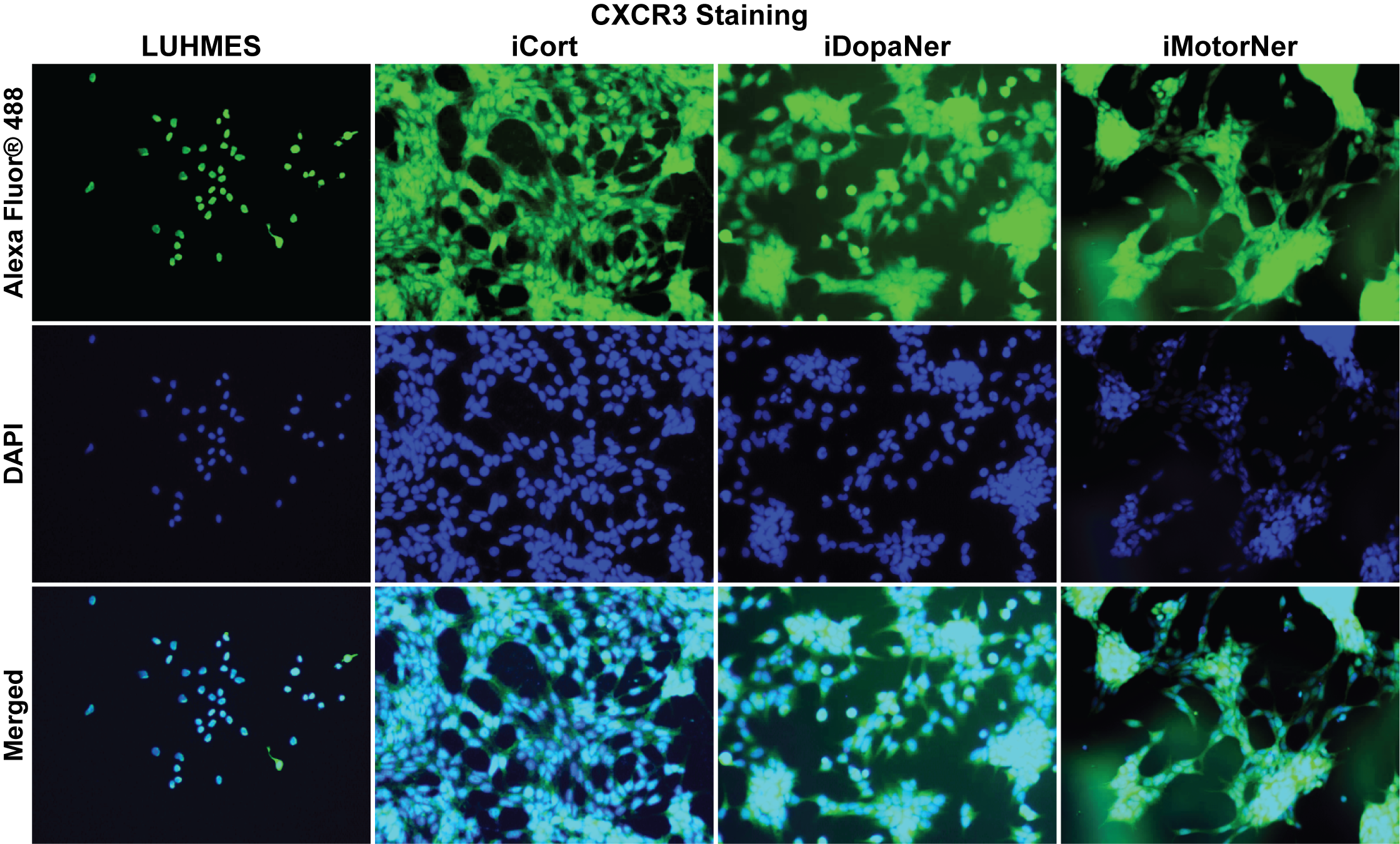

Supplement: S6 Fig — LUHMES- and iPSC-derived neurons were stained with antibody against CXCR3 (green). Alexa Fluor 488 anti-rabbit was used as secondary antibody. DAPI (blue) indicates nuclear staining. (TIF) [file ppat.1008249.s006.tif]

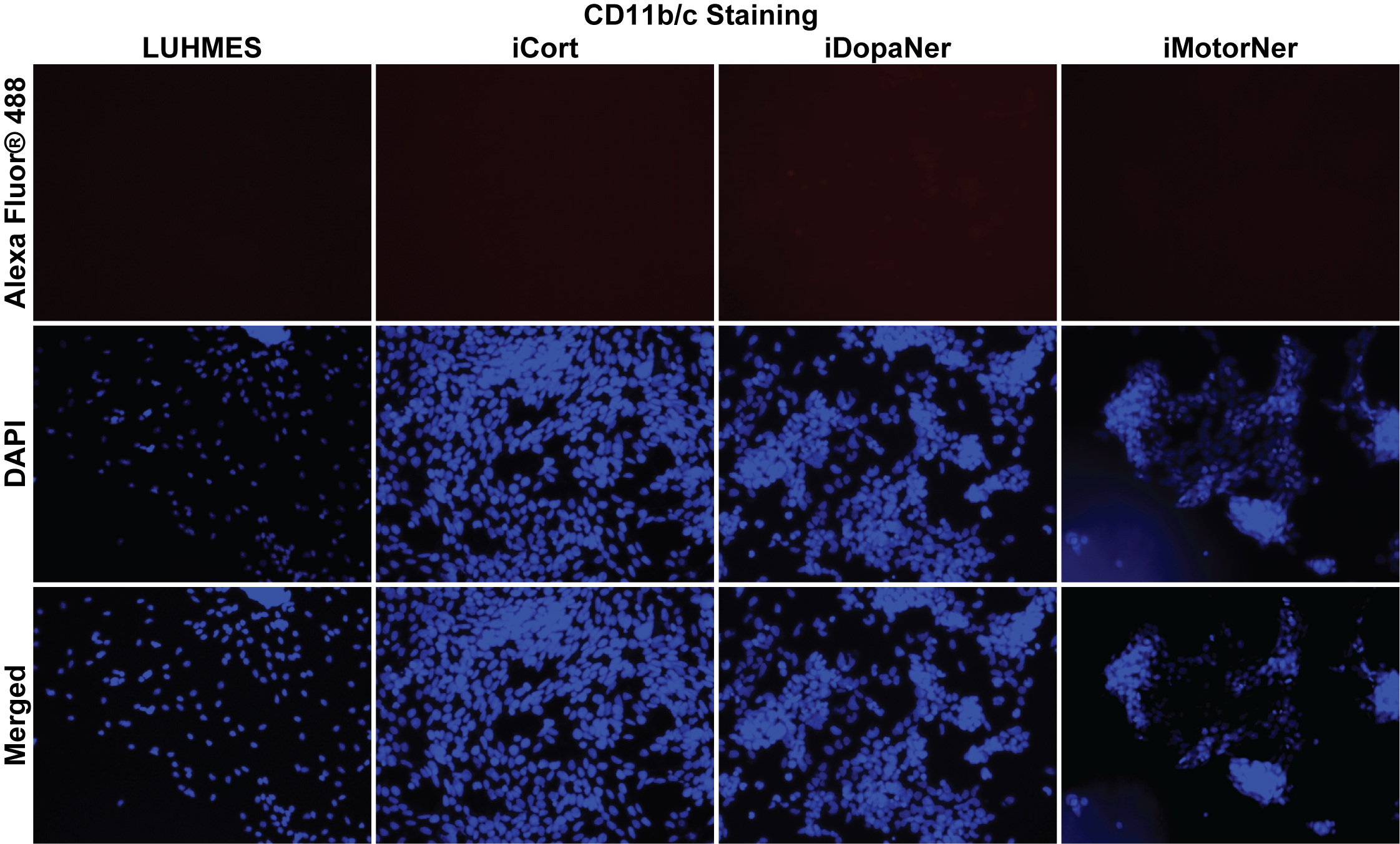

Supplement: S7 Fig — LUHMES- and iPSC-derived neurons were stained with antibody against CD11b/c. Alexa Fluor 488 anti-rabbit was used as secondary antibody. DAPI (blue) indicates nuclear staining. (TIF) [file ppat.1008249.s007.tif]

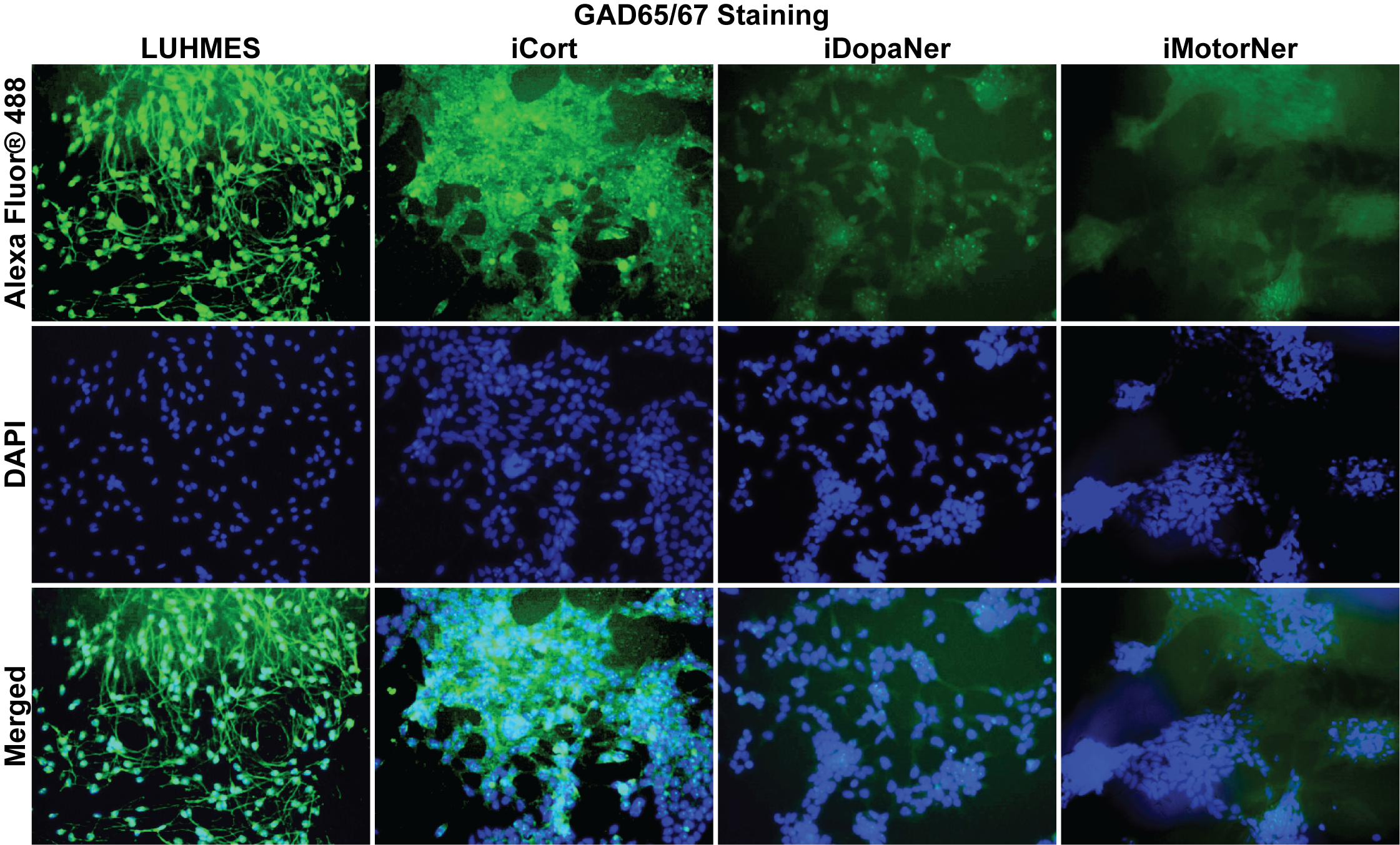

Supplement: S8 Fig — LUHMES- and iPSC-derived neurons were stained with antibody against GAD65/67 (green). Alexa Fluor 488 anti-rabbit was used as secondary antibody. DAPI (blue) indicates nuclear staining. (TIF) [file ppat.1008249.s008.tif]

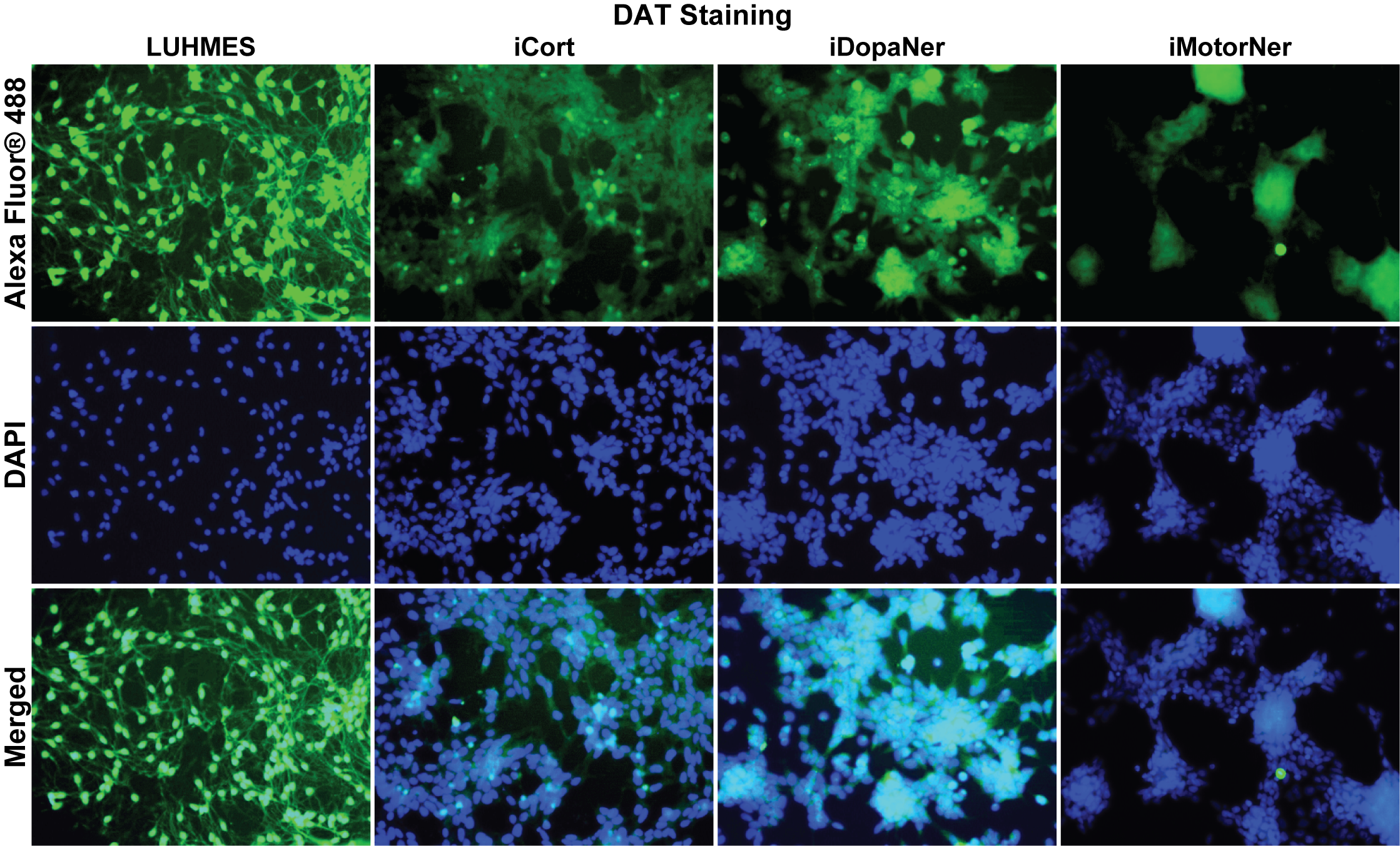

Supplement: S9 Fig — LUHMES- and iPSC-derived neurons were stained with antibody against DAT (green). Alexa Fluor 488 anti-rabbit was used as secondary antibody. DAPI (blue) indicates nuclear staining. (TIF) [file ppat.1008249.s009.tif]

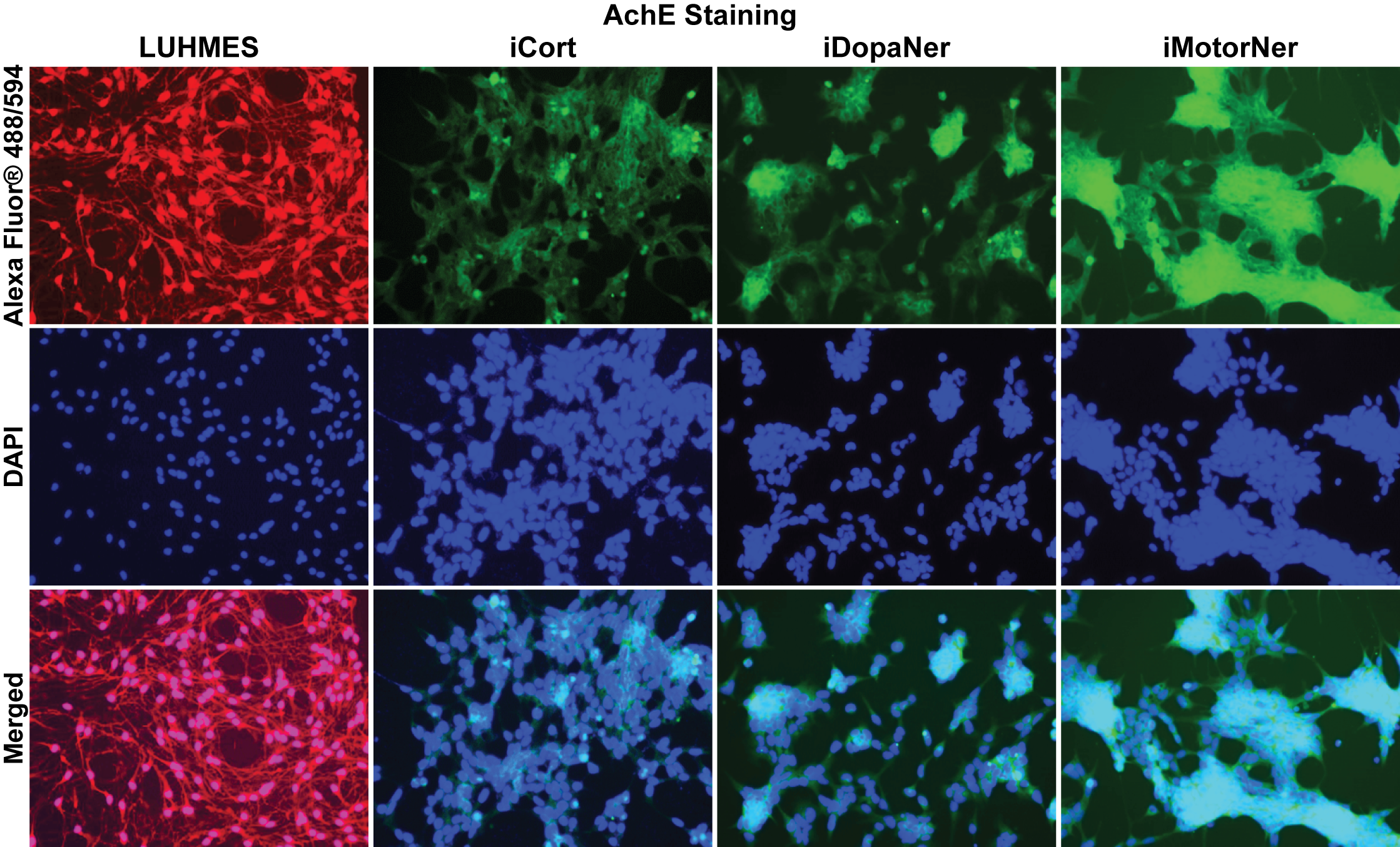

Supplement: S10 Fig — LUHMES- and iPSC-derived neurons were stained with antibody against AchE (red or green). Alexa Fluor 488 anti-rabbit (green) or Alexa Fluor 594 anti-mouse was used as secondary antibodies. DAPI (blue) indicates nuclear staining. (TIF) [file ppat.1008249.s010.tif]

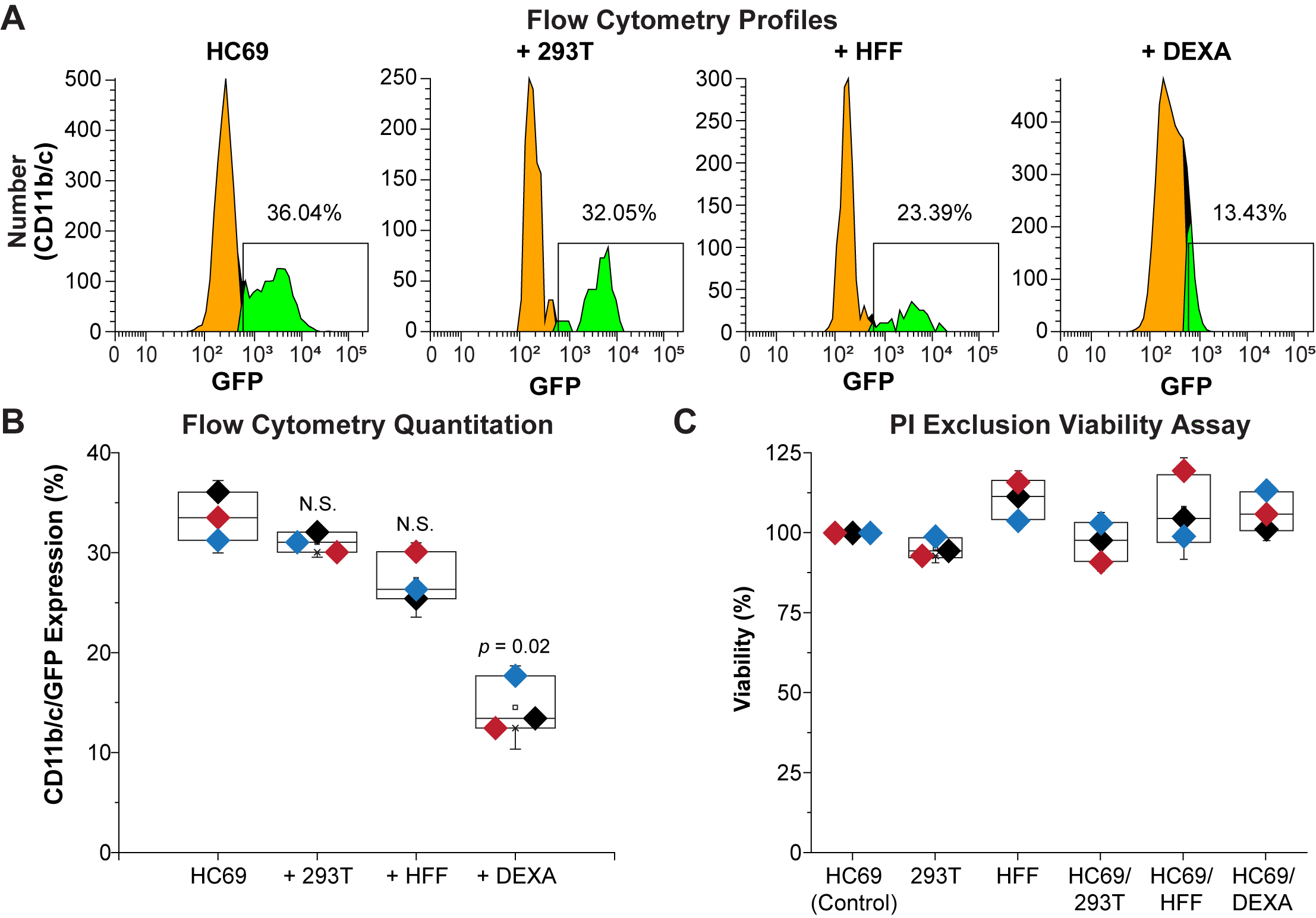

Supplement: S11 Fig — (A) 60,000 hμglia/HIV HC69 cells were plated in the absence or presence of 0.5 x 10^6^ 293T cells or human foreskin fibroblasts (HFF), both grown in DMEM/10% FBS, or DEXA (positive control). The co-culture medium was the immortalized microglia medium (Table 2). HIV expression was evaluated after 24 h by flow cytometry. Flow cytometry profiles representing single cultures indicate the proportion of the CD11b/c-expressing cells (total microglia; Y-axis) that expresses GFP (X-axis). (B). Flow cytometric analysis quantification of microglial cell GFP expression in three similar experiments. The p-values of pair-sample, Student’s t-tests comparing the microglial cells cultured alone or in the presence of cells are shown. Individual independent experiments are color coded (n = number of independent samples). N.S.: non-significant. (C) PI exclusion assay to evaluate co-culture viability. Viability values (Y-axis) were normalized to the control culture of HC69 cells alone. Each colored symbol represents one experiment. (TIF) [file ppat.1008249.s011.tif]

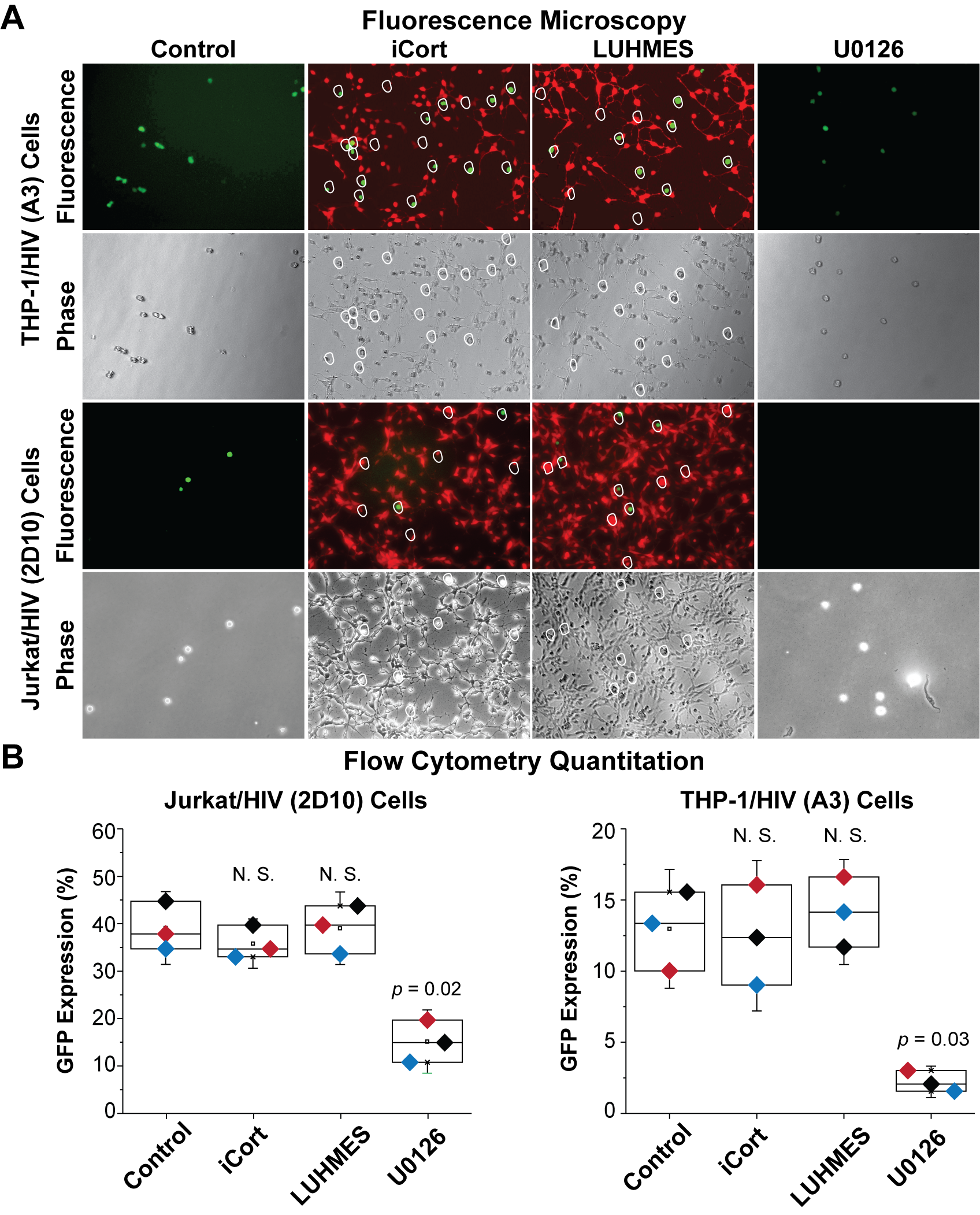

Supplement: S12 Fig — 60,000 THP-1/HIV (A3) and Jurkat/HIV (2D10) cells, grown as previously described [37], were plated in the absence or presence of 0.5 x 10^6^ iCort or LUHMES-derived neurons, or U0126 (positive control). NDM (Table 2) was co-culture medium. HIV expression was evaluated after 24 h by fluorescence microscopy (A) and flow cytometry. (B). Flow cytometric analysis of GFP expression in three similar experiments: the p-values of pair-sample, Student’s t-tests comparing the A3 and 2D10 cells cultured alone or in the presence of neurons are shown. Individual independent experiments are color-coded (n = number of independent samples). N.S.: non-significant. (TIF) [file ppat.1008249.s012.tif]

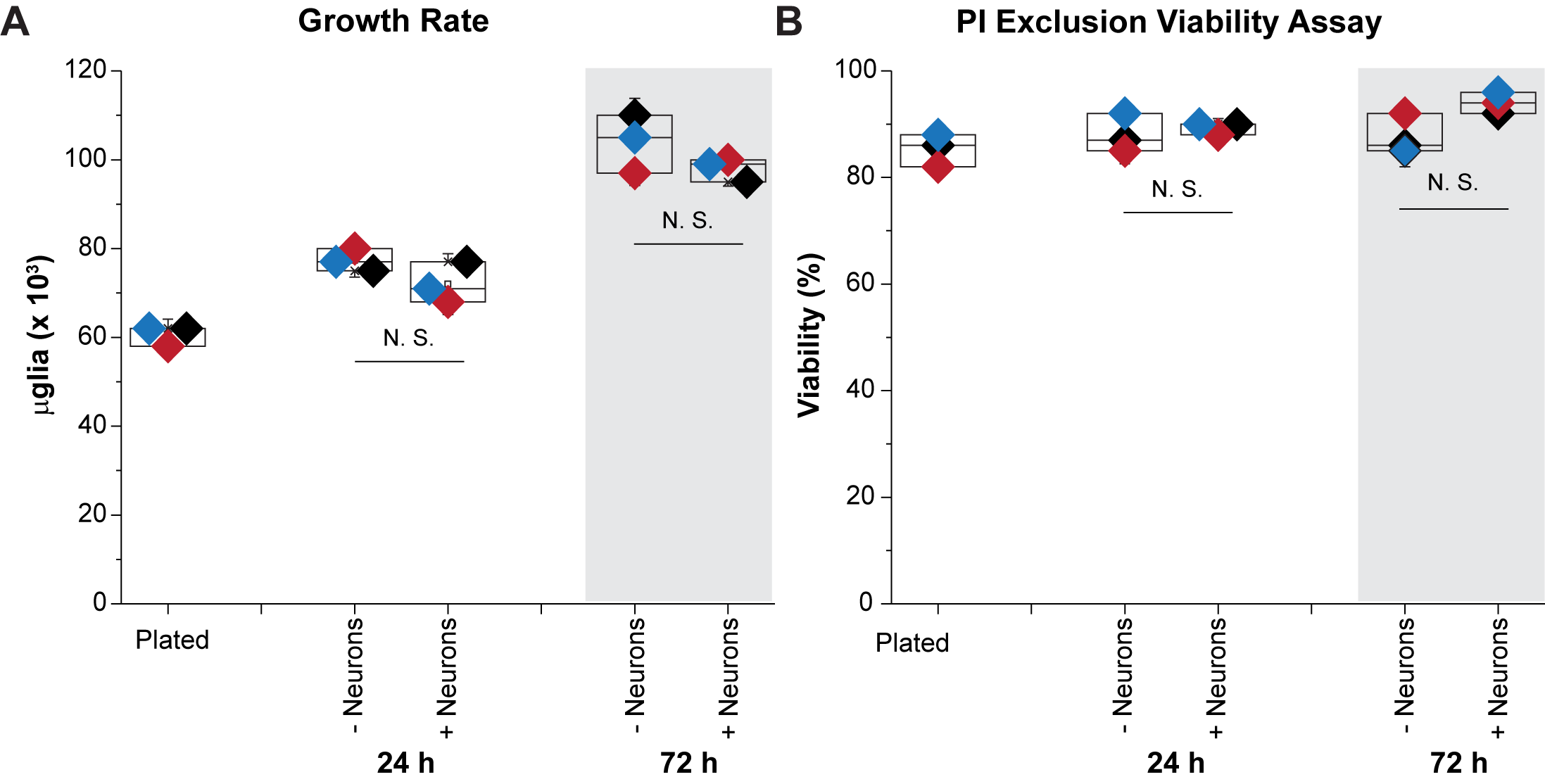

Supplement: S13 Fig — (A) Growth rate. 60,000 hμglia/HIV HC69 cells were plated in the presence of 0.5 x 10^6^ LUMHES-derived neurons (X-axis). After either 24 h or 72 h, neurons were killed with 0.25% trypsin for 30 seconds, and washed away with PBS prior to further trypsinization for 5 minutes to recover microglial cells. Cells were counted (Y-axis; left panel). (B) PI exclusion assay for measuring viability (Y-axis; right panel). N.S.: not significant. (TIF) [file ppat.1008249.s013.tif]

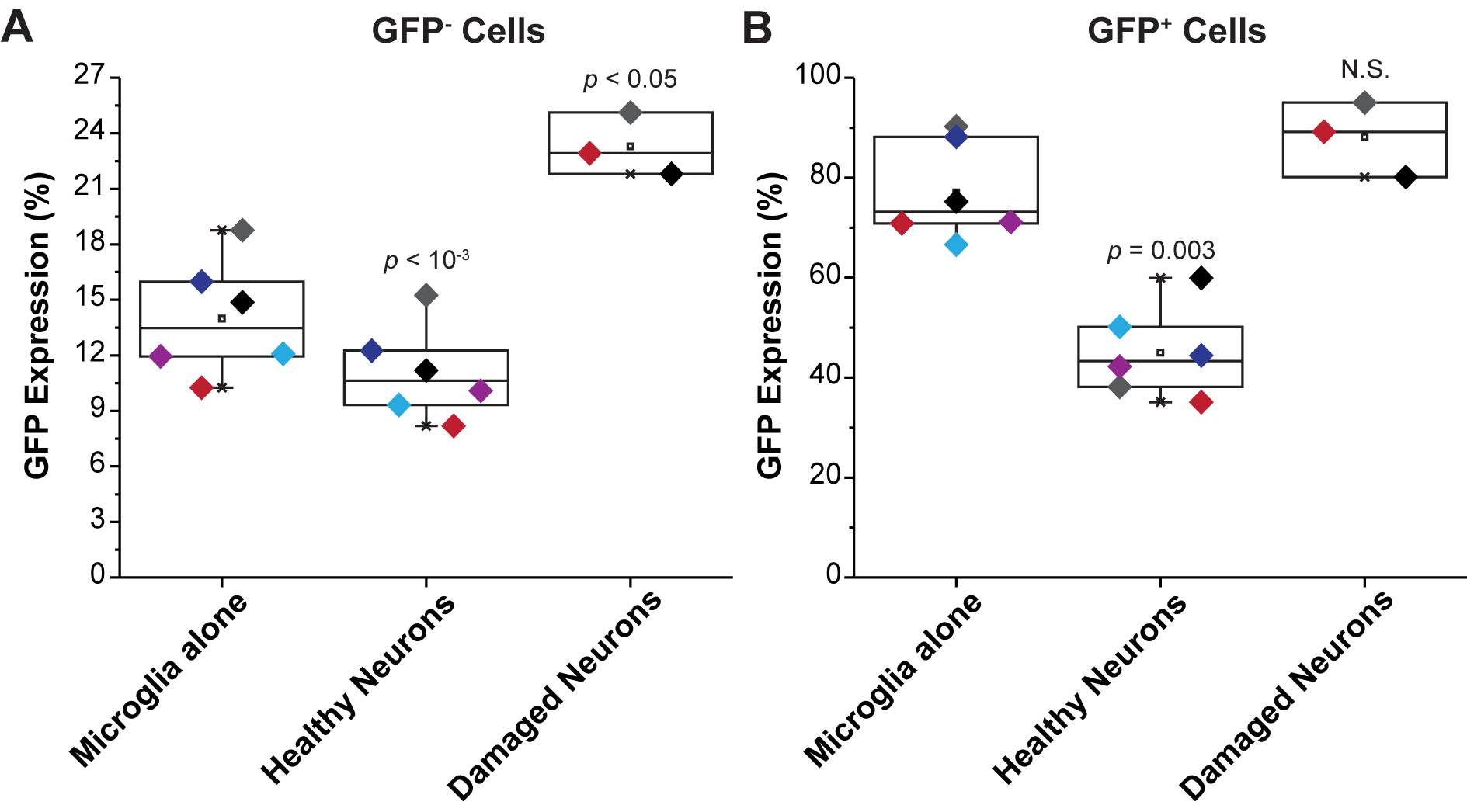

Supplement: S14 Fig — (A) hμglia/HIV HC69 cells were sorted into GFP- cells. The population was expanded for 48 h prior to collection and co-cultured with either healthy neurons or damaged neurons (X-axis) at a ratio of 50:6. (B) GFP+ cells. Quantitation of GFP expression (Y-axis). Diamonds of similar color represent an individual experimental series. (n = number of individual samples). The p-values of pair-sample t-tests comparing the unexposed vs. the exposed cells are shown. N.S.: non-significant. (TIF) [file ppat.1008249.s014.tif]

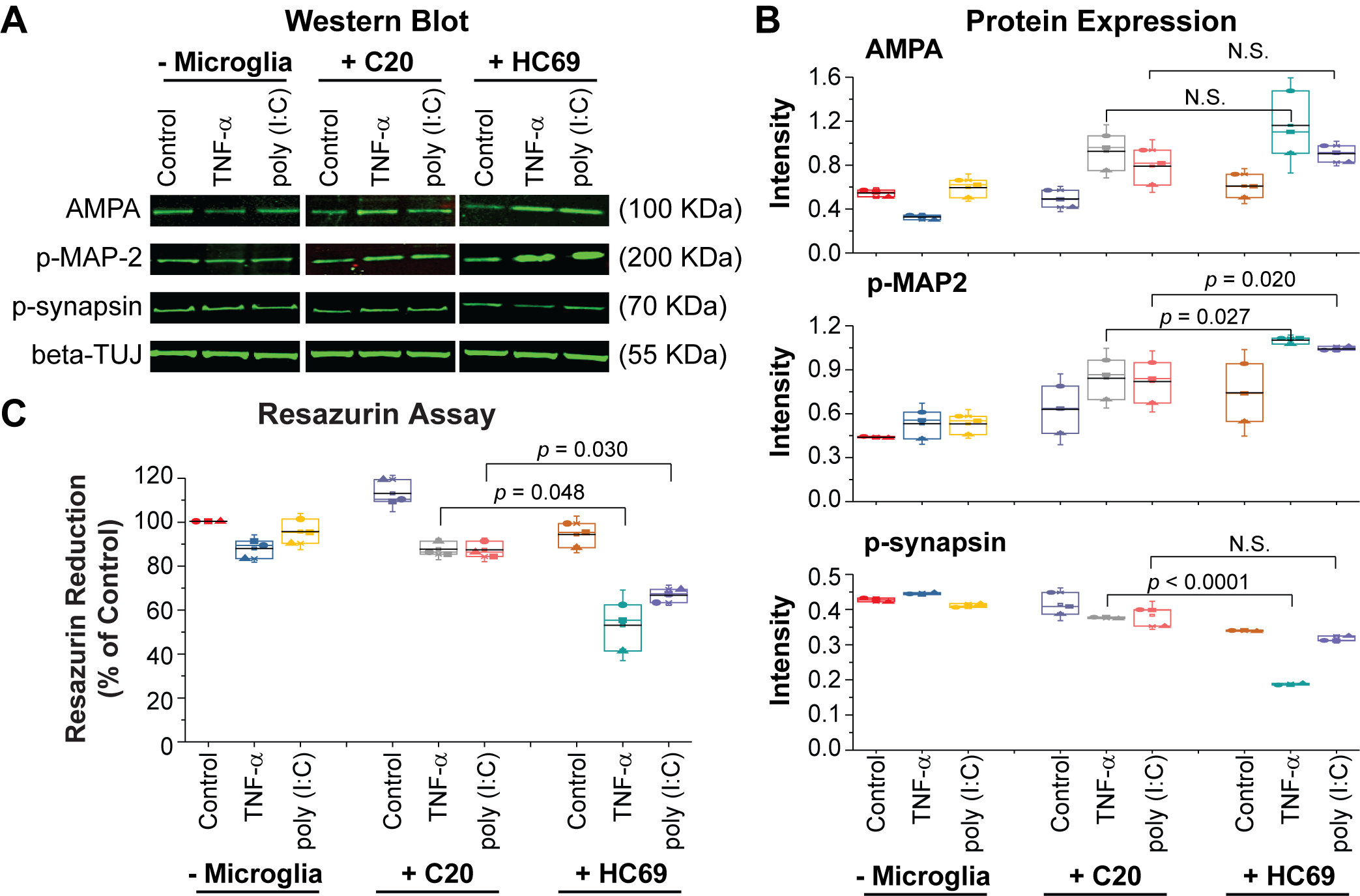

Supplement: S15 Fig — A. Western blot analysis. LUHMES-derived neurons were unexposed (-Microglia) or exposed to either C20 or HC69 cells in the absence or presence of either TNF-α or poly(I:C) for 48 h. Whole cell extracts were prepared from neurons and subjected to SDS-PAGE/Western blot analysis. Western blot membranes were blotted against anti-AMPA, anti-p-MAP2, and anti-p-synapsin antibodies, using anti-β-tubulin III as loading control. Approximate molecular weights are indicated in KDa. B. Quantitation of AMPA, p-MAP2, and p-synapsin band intensity. Numbers were plotted in Relative Intensity (Arbitrary Units; Y-axis) vs. experimental treatments (represented by a specific color; X-axis), graphs for each AMPA, p-MAP2, and p-synapsin. C. Resazurin reduction assay. The resazurin reduction values (Y-axis) plotted are referenced to the control culture (neurons only), set at 100%, next to the other experimental treatments (X-axis). For (B) and (C), the p-values of statistically significant pair-sample t-tests (at the 0.05 confidence level, where the difference of the sample means is significantly different from the test difference of zero) of three experiments (n = 3) comparing the neurons exposed to C20 and either TNF-α or poly(I:C) with the neurons exposed to HC69 and either TNF-α or poly (I:C), respectively. N.S. stands for non-significant. Similar geometric figures represent a unique experiment. (TIF) [file ppat.1008249.s015.tif]

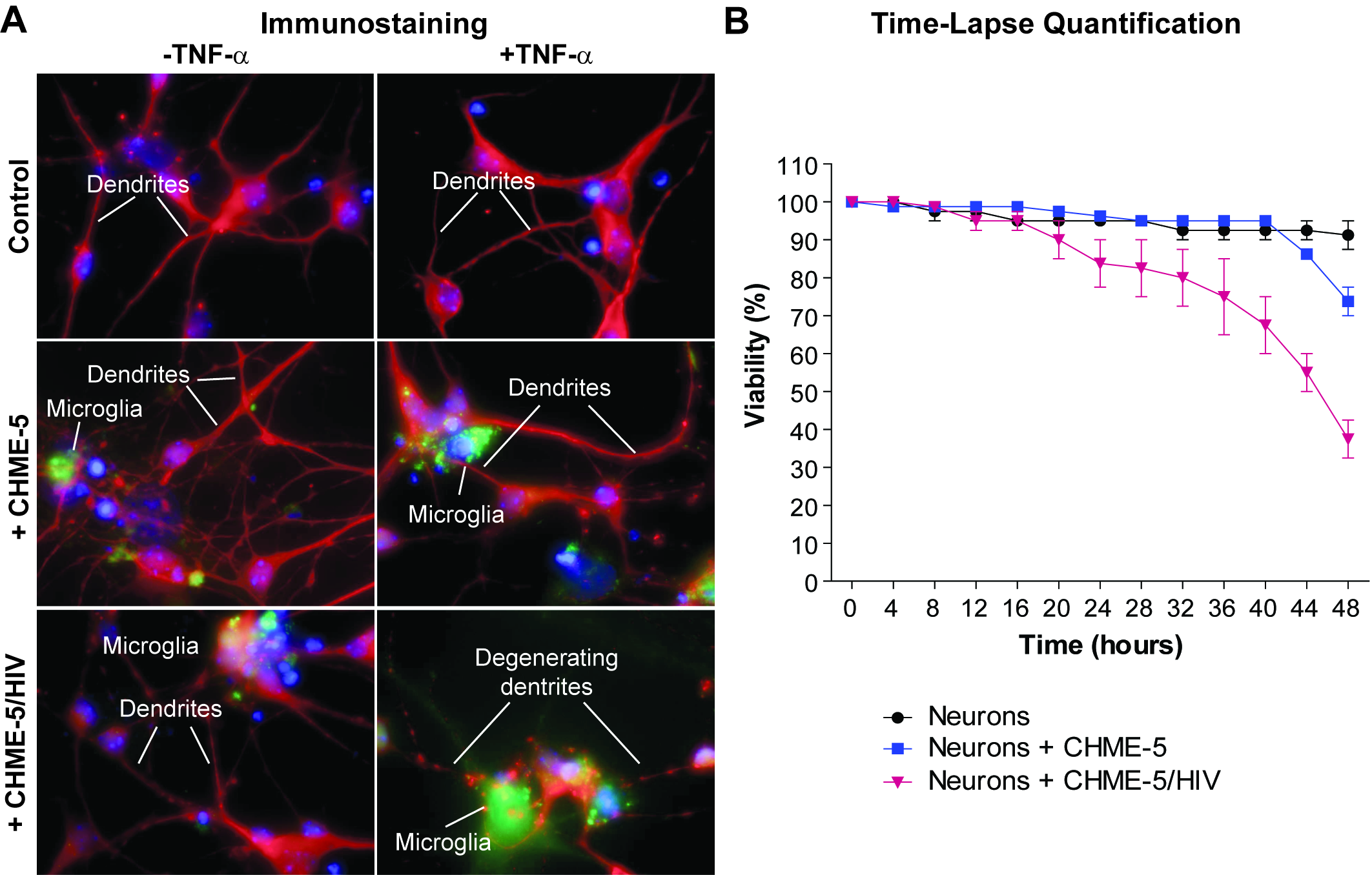

Supplement: S16 Fig — A. LUHMES-derived neurons were co-cultured or not with CHME-5 or CHME-5/HIV cells for 48 h. TNF-α (100 ng/mL) was added or not, as indicated. The neurons or co-cultures were stained with anti-MAP2 antibody followed by anti-rabbit Alexa Fluor 594 antibody (red) and DAPI for nuclear visualization. Green (GFP) depicts activated CHME-5/HIV cells. Dendrites and microglia are indicated by the white lines. B. LUHMES-derived neurons were co-cultured with CHME-5 (blue squares) or CHME-5/HIV (red triangles) cells that had been pre-activated with 100 ng/mL of TNF-α. Neurons alone (black circles) were used as control. Time-lapse images were taken every four hours, at the indicated time points, from 0 to 48 h (X-axis). The number of healthy neurons was counted in every field the relative number of viable neurons quantified (Y-axis). (TIF) [file ppat.1008249.s016.tif]

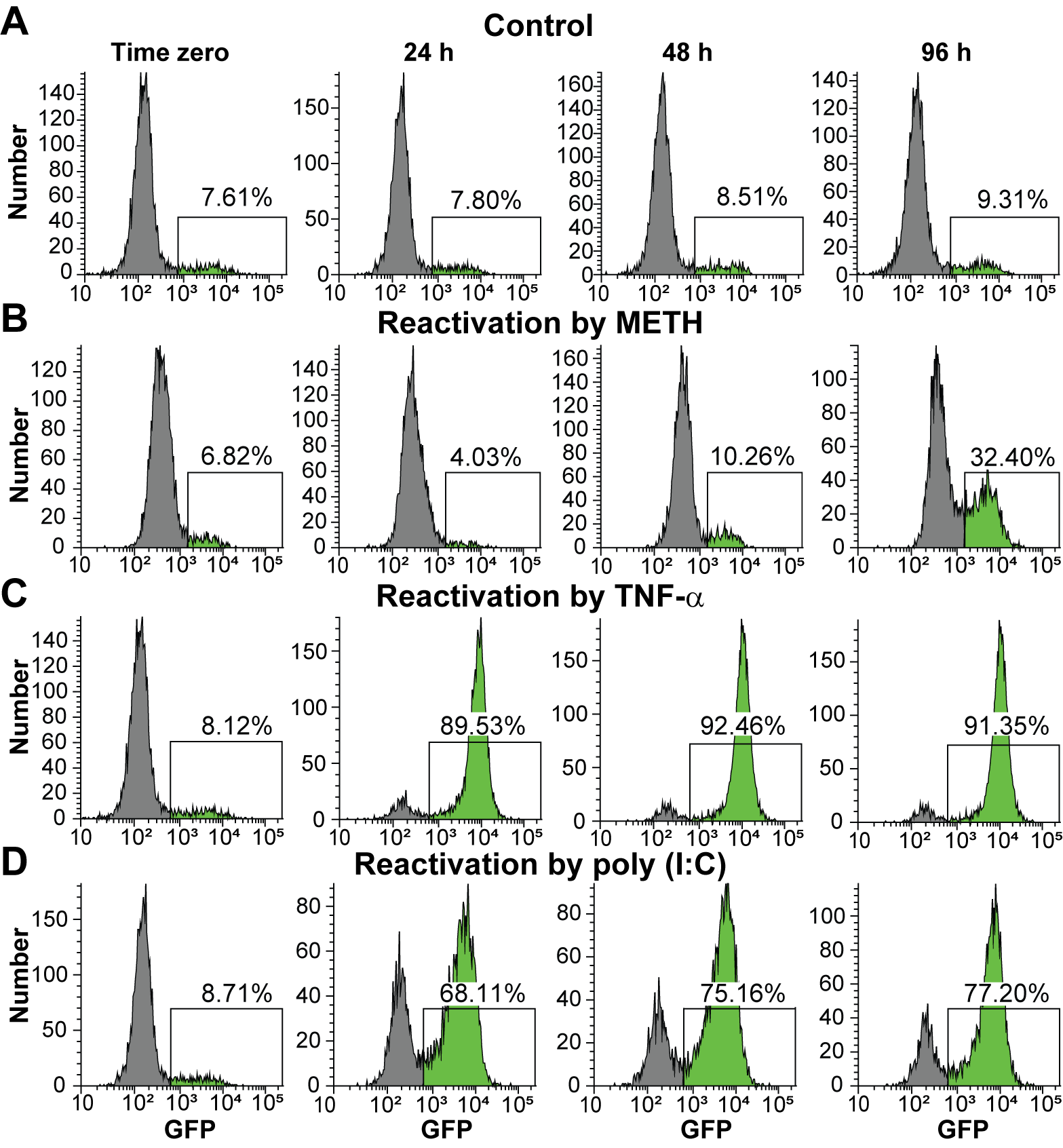

Supplement: S17 Fig — Flow cytometry profiles representing single cultures of HC69 cells were incubated for 24, 48 or 96 h. (A) Untreated. (B) 300 μM METH. (C) 100 pg/ml TNF-α. (D) 100 ng/mL poly (I:C). GFP+ cell populations were measured by flow cytometry and indicated in bright green. (TIF) [file ppat.1008249.s017.tif]

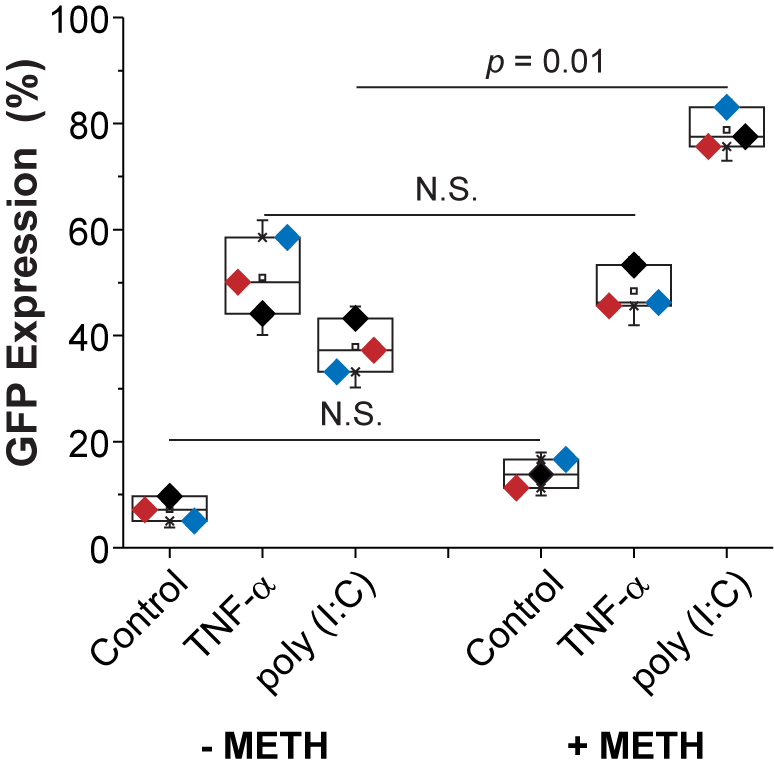

Supplement: S18 Fig — HC69 cells were either untreated (- METH) or treated with METH 300 μM for 72 h prior to exposure to either TNF-α 20 pg/mL) or poly(I:C) (50 ng/mL) for another 24 h. Diamonds of similar color represent an individual experimental series. (n = number of individual samples). The p-values of pair-sample t-tests comparing the unexposed vs. the exposed cells are shown. N.S.: non-significant. (TIF) [file ppat.1008249.s018.tif]

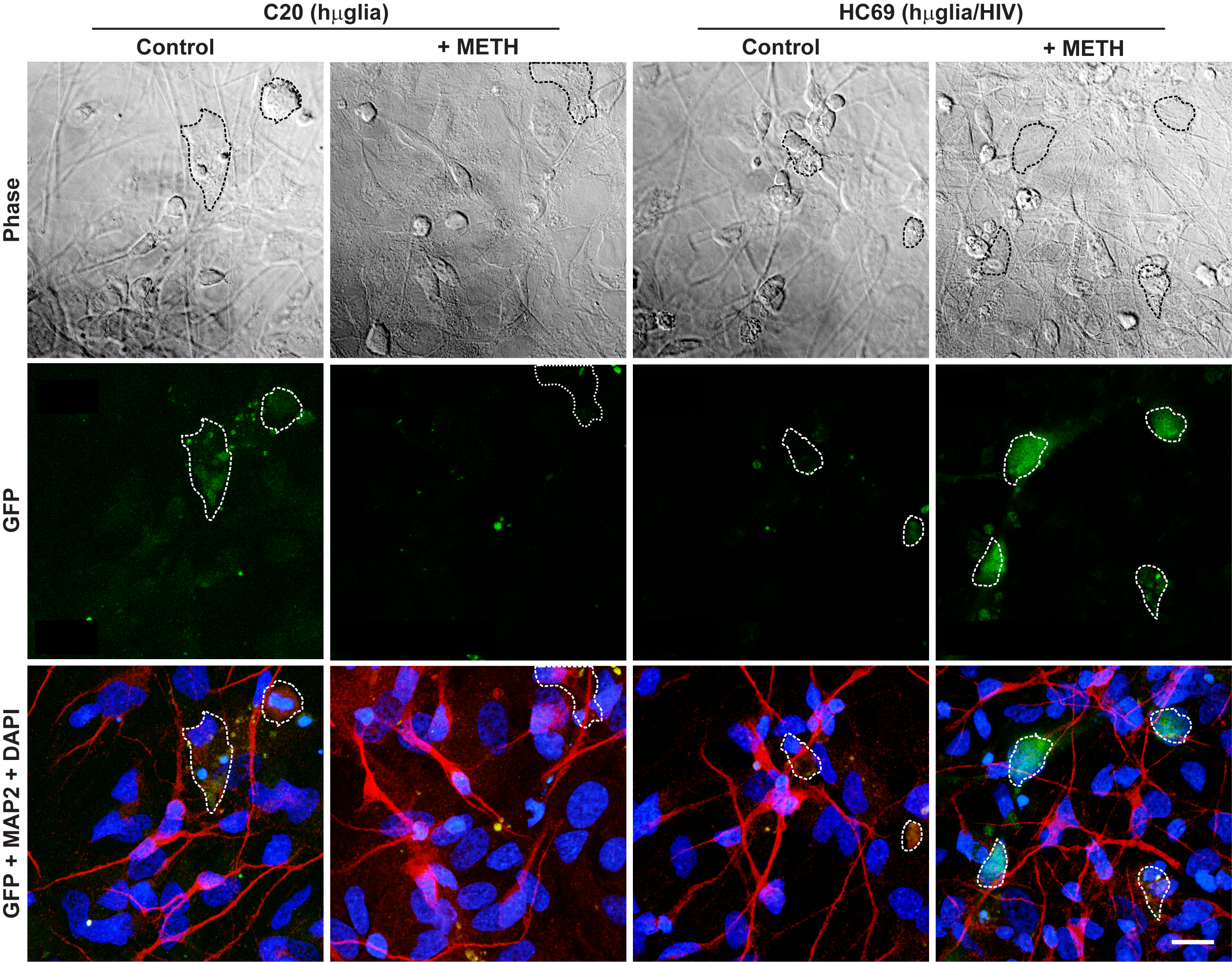

Supplement: S19 Fig — Human neuronal and glial mixed-cultures containing astrocytes (Advanced Biosci. Resources) were maintained for 17 days in vitro (DIV) in BrainPhys supplemented with insulin-transferrin-sodium selenite prior to co-culture with either C20 or HC69 cells in either the absence or presence of 300 μM METH for 72 h. Top: brightfield. Middle: Green fluorescence channel. Bottom: Green (GFP+ cells). Red (MAP2, neuronal dendrites). Blue (DAPI, all nuclei). (TIF) [file ppat.1008249.s019.tif]

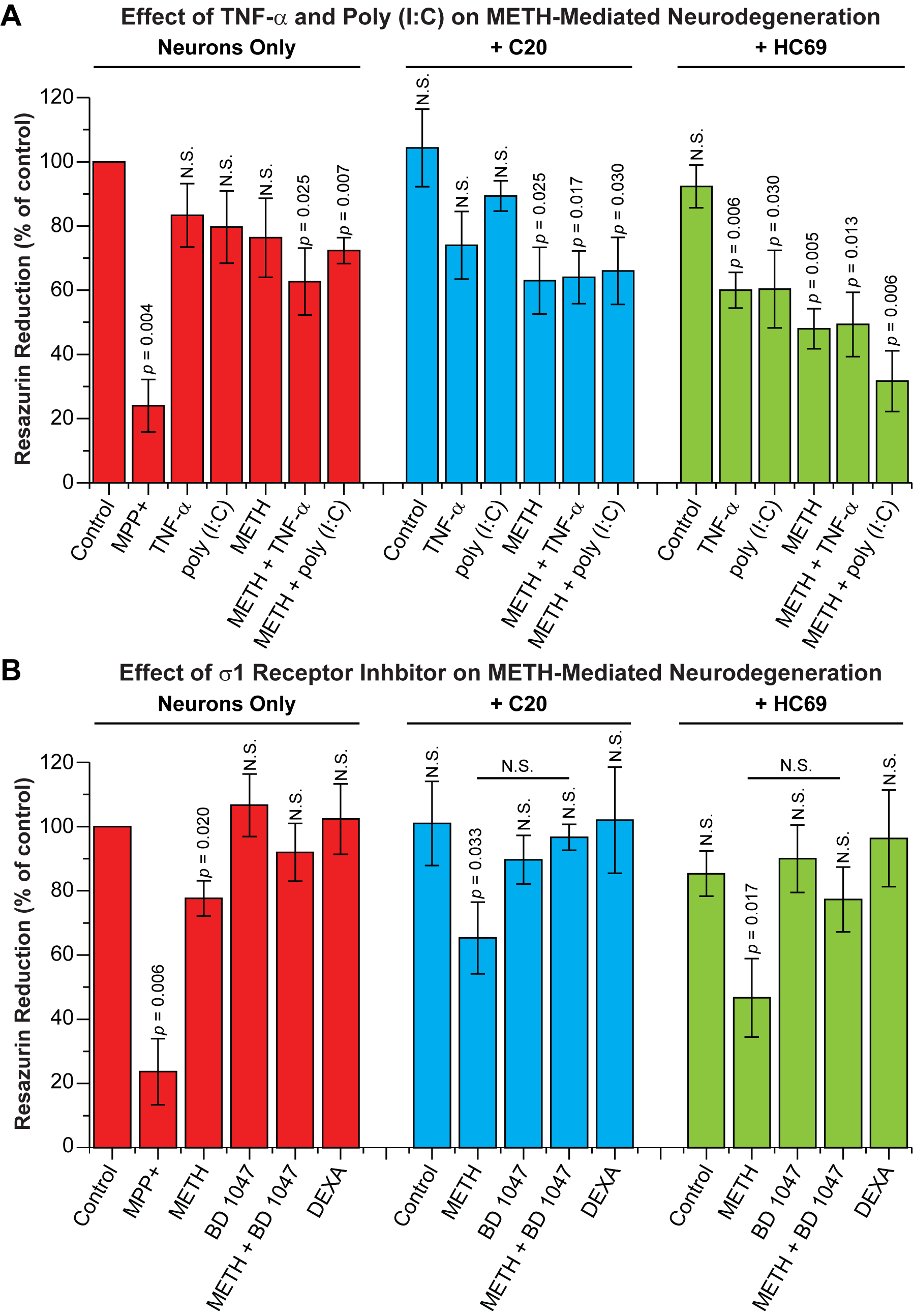

Supplement: S20 Fig — LUHMES-derived neurons were either cultured alone (red) or co-cultured with either C20 (blue) or HC69 (green) cells in either the absence (control) or presence of (A) TNF-α, poly (I:C), METH, METH + TNF-α or METH + poly (I:C), or (B) METH, BD1047, METH + BD1047 or DEXA for 72 h (X-axis) prior to neuronal survival quantitation by the resazurin method (Y-axis). MPP+ was used as positive control for neuronal damage. (TIF) [file ppat.1008249.s020.tif]
